# A Retrospective Evaluation of Ototoxicity Monitoring in a Cohort of Pediatric Patients With Solid Tumors, Treated in the Dutch National Cancer Center

**DOI:** 10.1002/cnr2.70046

**Published:** 2024-11-26

**Authors:** Franciscus A. Diepstraten, Odette M. M. Bertram, Hiske W. Helleman, Ralf A. Boerboom, Martine van Grotel, József Zsíros, Godelieve A. M. Tytgat, Harm van Tinteren, Robert J. Stokroos, Geert O. Janssens, Alex E. Hoetink, Marry M. van den Heuvel‐Eibrink, Annelot J. M. Meijer

**Affiliations:** ^1^ Princess Máxima Center for Pediatric Oncology Utrecht Netherlands; ^2^ Department of Audiology University Medical Center Utrecht Utrecht Netherlands; ^3^ Department of Otorhinolaryngology‐Head and Neck Surgery University Medical Center Utrecht Utrecht Netherlands; ^4^ UMC Brain Center University Medical Center Utrecht Utrecht Netherlands; ^5^ Department of Radiation Oncology University Medical Center Utrecht Utrecht Netherlands; ^6^ University Medical Center Utrecht‐Wilhelmina Children's Hospital Utrecht Netherlands

**Keywords:** audiology, childhood cancer, ototoxicity, solid tumors

## Abstract

**Introduction:**

Ototoxicity is an adverse effect of childhood cancer treatment with a negative impact on speech‐language development and quality of life. This study aimed to retrospectively assess ototoxicity monitoring in a national cohort of pediatric patients with solid tumors, examining the frequency and determinants associated with hearing loss (HL).

**Methods:**

This retrospective cohort study included 305 patients treated between 2015 and 2020 at the Princess Máxima Center. Patients receiving platinum agents, head and neck radiotherapy, and/or ear‐nose‐throat surgery were analyzed. Electronic patient files provided demographic, clinical, and audiological data. HL was defined as Muenster ≥ 2b or SIOP ≥ 2 grade. Associations between clinical characteristics and HL occurrence were analyzed using logistic regression analysis.

**Results:**

Audiological monitoring was performed at baseline (62.6%), during treatment (79.0%), and at the end of treatment (82.1%). Post treatment, 51.2% and 36.5% experienced Muenster and SIOP‐defined HL, respectively. Multivariable analyses revealed that age at diagnosis (OR 0.9, 95% CI 0.9–1.0), total cumulative dose cisplatin per 100 mg/m^2^ (OR 1.6, 95% CI 1.4–2.0), and vincristine treatment (OR 3.3, 95% CI 1.4–7.8) remained significantly associated with Muenster grade ≥ 2b HL. Age at diagnosis in years (OR 0.9, 95% CI 0.8–1.0), total cumulative dose cisplatin per 100 mg/m^2^ (OR 1.5, 95% CI 1.2–1.8), and male sex (OR 2.7, 95% CI 1.4–5.3) were associated with SIOP ≥ 2 HL.

**Conclusion:**

This study shows that more than half of the children treated with ototoxic cancer therapies develop HL by the end of treatment. Therefore, audiological monitoring during and after treatment is essential. Improved insight into clinical determinants aids in identifying patients at high risk for HL, who may benefit from prevention strategies that are currently being implemented.

**Abbreviations**
BERA: brainstem‐evoked response audiometry
CI: confidence interval
CNS: central nervous system
CPA: conditioned play audiometry
DPOAE: distortion product otoacoustic emissions
EHF: extended High frequencies
ENT: ear nose throat
H&N‐RT: head and neck radiotherapy
HL: hearing loss
kHz: kilo Hertz
OR: odds ratio
PMC: Princess Máxima Center
PTA: pure‐tone audiometry
SIOP: International Society for Pediatric Oncology
TEOAE: transiently evoked otoacoustic emissions
VRA: visual reinforcement audiometry
WKZ: Wilhelmina Children's Hospital

## 1 Introduction

In the Netherlands, about 600 children are being diagnosed with cancer each year [1]. Due to advancements in diagnostic modalities and novel treatment strategies over the past decades, the overall survival of these children has increased to around 80% in high‐income countries [2]. As more children survive cancer, reducing the serious treatment‐related adverse events has become increasingly important. One of the most frequently occurring early adverse events is ototoxicity, characterized by hearing loss (HL), tinnitus, and/or vertigo, [3] which can have a negative impact on the child's life [4]. Reported prevalence rates of HL differ between studies, ranging from 1.7% to 69.1% depending on the sample size, specific disease, cancer treatment, and ototoxicity classifications used [5].

Several studies have identified clinical risk factors associated with HL. Treatment with platinum‐based chemotherapy (cisplatin, carboplatin), cranial irradiation, and central nervous system (CNS) surgery have been proven to cause irreversible ototoxicity [6, 7, 8, 9]. Research has also demonstrated that supportive care medication such as aminoglycosides, glycopeptides, and loop diuretics contribute to the development of HL [10, 11, 12, 13]. Furthermore, the ototoxic (additional) effect of vincristine chemotherapy has recently been reported [14, 15].

Although international cancer treatment protocols include recommendations for audiological monitoring in children with cancer (Table S1), adherence to these protocols in clinical practice remains challenging [16]. A population‐based study among Swiss childhood cancer survivors reported that less than half of the patients at risk for ototoxicity had been subject of audiological monitoring before, during and after ototoxic treatment [17]. To increase adherence in clinical practice, in 2021, experts in the field of pediatric oncology and audiology developed a consensus guideline, with the overall recommendation to perform age‐appropriate testing before and at the end of treatment as a minimum, and to consider testing during treatment if possible [18].

In this retrospective study, we present the results on ototoxicity monitoring in the first 5 years (2015–2020) of the Dutch national childhood cancer center, the Princess Máxima Center. The first aim is to reveal which proportion of patients were referred for audiological monitoring according to their treatment protocol. Furthermore, the prevalence of HL in this cohort is unknown. The results will provide insight into the referral pattern, frequency, and determinants of HL in children with cancer.

## 2 Methods

### 2.1 Study Population and Study Design

In this retrospective cohort study, the following inclusion criteria were applied: 1. pediatric patients with cancer diagnosed with a solid tumor; 2. age between 0 and 19 years at time of diagnosis; 3. diagnosis and start cancer treatment in the Máxima between January 1, 2015, and December 31, 2020, according to (inter)national cancer treatment guidelines; and 4. treatment with platinum chemotherapy, ear‐nose‐throat (ENT) surgery and/or head and neck radiotherapy (H&N‐RT). Hence, patients were excluded if they: 1. started initial treatment before 2015 or after 2020; 2. were initially treated before 2015 and had a recurrence or relapse of disease between 2015 and 2020; 3. started cancer treatment in an international hospital, and 4. if they denied informed consent for inclusion in the Máxima Biobank. The study was approved by the Máxima Biobank and Data Access Committee (PMCLAB2021.275).

### 2.2 Data Collection

Clinical, demographic, and audiological data were anonymously transferred and collected in a dashboard from electronic patient files, including age at diagnosis, sex, solid tumor type, applied (inter) national cancer treatment protocol, start and end date of treatment, and whether a patient had received treatment with ENT surgery, H&N‐RT, platinum agents (cisplatin, carboplatin and/or oxaliplatin), and/or vincristine. Data on supportive care medication, including aminoglycosides (gentamicin, amikacin, tobramycin), glycopeptides (vancomycin, teicoplanin), and diuretics (amlodipine, furosemide), were extracted. Audiological variables consisted of the type of audiological test (otoscopy, tympanometry), transiently evoked otoacoustic emissions [TEOAE], distortion product otoacoustic emissions [DPOAE], brainstem‐evoked response audiometry [BERA], visual reinforcement audiometry [VRA], conditioned play audiometry [CPA], pure‐tone audiometry [PTA], extended high frequency [EHF] PTA, and the results of these tests. In case audiological tests were not performed in patients at risk for ototoxicity, the electronic patient files were checked for an explanation for the absence of an audiological measurement. Audiological data was made available by the Audiology Department of the Wilhelmina Children's Hospital (WKZ).

### 2.3 Definition of HL

HL was specified using the Muenster classification [19], in which grade 2b (> 40 dB HL at ≥ 4 kHz or above, i.e., 6 or 8 kHz) or higher in at least one ear was defined as deleterious HL. The SIOP ototoxicity scale [20] was used as a secondary classification for sensorineural HL, in which deleterious HL was defined as > 20 dB at 4 kHz and above (corresponding with grade 2) (Table S2) in at least one ear. To obtain a grading, audiometric PTA or CPA thresholds are required. Ear‐specific measurements for CPA/PTA were available, as masking of the nontested ear is routinely performed at our center. In younger or more difficult to test children, obtaining reliable thresholds with PTA or CPA is challenging and may not always be possible. In this subcohort of children, hearing function was measured by BERA and VRA in combination with OAEs, and the results were assessed by experienced audiologists (AH, HH, and RB). An audiological test was considered reliable when the results were reproducible and the child was cooperative during the audiological examination. Hearing function was categorized as sufficient for normal perception of speech, abnormal hearing (indicative for Muenster ≥ 2b and SIOP ≥ 2), or not conclusive. A summary of the applied derivative grading approach for BERA, VRA in combination with OAEs can be found in Table 1. Asymmetry of HL was reported and defined as a difference of ≥ 2 Muenster grades between the left and right ear. Mild ototoxicity, Muenster > 0 and < 2b and SIOP > 0 and < 2, was not specifically reported.

**TABLE 1:** Summary of applied derivative grading approach for BERA and VRA in combination with OAE measurements.

Test/measurement	Criteria	Derivative SIOP classification	Derivative muenster classification
Masked click BERA	Wave JV threshold—10 dB; estimation mainly for 3–4 kHz range.	≤ 20 dB: SIOP < 2	≤ 20 dB and DPOAEs present 6–8 kHz: Muenster < 2b
	JV latency shift indicates high frequency loss.	> 20 dB: SIOP ≥ 2	> 20 dB and/or abnormal DPOAEs 6–8 kHz: Muenster ≥ 2b
VRA in free field	Thresholds for 0.25–4 kHz; lowest stimulus level 20 dB (HL).	4 kHz threshold ≤ 20 dB: SIOP < 2	4 kHz threshold ≤ 20 dB and DPOAEs present 6–8 kHz: Muenster < 2b
		4 kHz threshold > 20 dB: SIOP ≥ 2	4 kHz threshold > 20 dB and/or abnormal DPOAEs 6–8 kHz: Muenster ≥ 2b
VRA with headphones	Ear‐specific thresholds for 0.25–4 kHz; lowest stimulus level 10–15 dB (HL).	4 kHz threshold ≤ 20 dB: SIOP < 2	4 kHz threshold ≤ 20 dB and DPOAEs present 6–8 kHz: Muenster < 2b
		4 kHz threshold > 20 dB: SIOP ≥ 2	4 kHz threshold > 20 dB and/or abnormal DPOAEs 6–8 kHz: Muenster ≥ 2b
TE‐ and DP‐OAEs	Used to refine classification when combined with BERA and VRA results. Always in combination with tympanometry. In isolation not usable for grading.		

Abbreviations: BERA, brainstem‐evoked response audiometry; dB, decibel; DPOAE, distortion product oto‐acoustic emissions; kHz, kilohertz; SIOP, international society for pediatric oncology; TEOAE, transiently evoked otoacoustic emission; VRA, visual reinforcement audiometry.

### 2.4 Statistical Analysis

Descriptive statistics were used to describe the cohort characteristics. Continuous variables were reported by using medians with ranges, and categorical variables were reported using numbers with percentages. Univariate and multivariate logistic regression analyses were performed after graphical inspection to analyze associations between clinical variables and presence of HL according to the Muenster and SIOP classification. These analyses were performed for the CPA/PTA subcohort and total cohort of children with interpretable end of treatment audiological evaluations. Multivariable logistic analysis applied a 10‐fold cross validation based on a training set consisting of 75% of the population and then evaluating (i.e., testing, validating) the performance of the model when applied to a test set of data (25%) drawn from the same population.

All variables with a *p* value of < 0.05 (2‐tailed) in the final model were considered statistically significant. Odds ratios (ORs) with 95% confidence intervals (CIs) were estimated.

Statistical analyses were performed using SPSS Statistics Version 26.0.0.1 (SPSS Inc., Chicago, IL, USA) [21] and R.

## 3 Results

### 3.1 Selection Process

**FIGURE 1:**
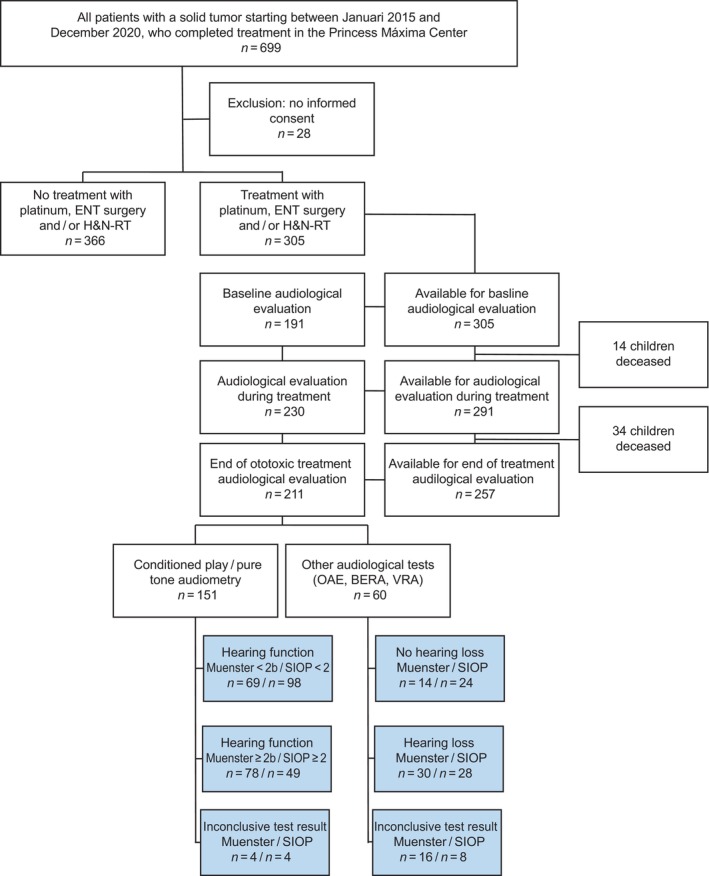
A flow chart describing the inclusion of patients, audiological tests, and results at the end of treatment. BERA, brainstem‐evoked response audiometry; OAE, oto‐acoustic emissions; VRA, visual reinforcement audiometry.

Between January 2015 and December 2020, 699 children with solid tumors were treated in the Máxima. 28 patients were excluded because they did not provide consent for inclusion in the Máxima Biobank. Of the remaining 671 patients, 305 (45.5%) patients were exposed to ototoxic treatment (Figure 1).

### 3.2 Characteristics of Study Population

In the entire study cohort of 305 children with different tumor types (Table 2), 53.1% were male. The median age at time of diagnosis was 4.0 years (range 0–18.0 years). Children were treated according to national and international treatment protocols. They received cisplatin only (59.0%), carboplatin only (17.4%), oxaliplatin only (0.3%) or a combination of platinum agents (18.4%). Furthermore, some of them received radiotherapy, with (0.3%) or without (1.6%) ENT surgery or with cisplatin (1.6%). Others received cisplatin and ENT surgery (1.3%) (Table 2). Median time between end of treatment and audiological assessment was 2.0 months (range − 2.9–78.8 months) and. 1.6 months (range − 11.6–17.2 months) for children tested with CPA/PTA and other tests, respectively (Table 3).

**TABLE 2:** Characteristics of the study cohort: Children treated with platinum agents, H&N‐RT and/or ENT‐surgery.

		Audiological evaluation after end of ototoxic treatment		
		Yes (*N* = 211)		No (*N* = 94)
	Total cohort	CPA/PTA subcohort	Other audiological test (OAE, BERA, VRA) subcohort	
	*N* = 305	*N* = 151	*N* = 60	*N* = 94
Sex, *n* (%)				
Male	162 (53.1)	80 (53.0)	32 (53.3)	50 (53.2)
Female	143 (46.9)	71 (47.0)	28 (46.7)	44 (46.8)
Median age at diagnosis in years (range) Percentiles	4.0 (0–18)	6.0 (0–18)	0.5 (0–13)	4.0 (0–18)
25	1.0	4.0	0.0	2.0
50	4.0	6.0	0.5	4.0
75	12.0	13.0	1.0	13.3
Cancer diagnosis, *n* (%)				
Neuroblastoma	119 (39.0)	55 (36.4)	28 (46.7)	36 (38.2)
Osteosarcoma	41 (13.4)	31 (20.5)	0 (0.0)	10 (10.6)
Ewing sarcoma	2 (0.7)	1 (0.7)	0 (0.0)	1 (1.1)
Germ cell tumor	46 (15.1)	23 (15.2)	12 (20.0)	11 (11.7)
Liver tumor	27 (8.9)	8 (5.3)	14 (23.3)	5 (5.3)
Renal tumor	43 (14.1)	18 (11.9)	4 (6.7)	21 (22.3)
Soft‐tissue sarcomas	7 (2.3)	5 (3.3)	0 (0.0)	2 (2.1)
Nasopharyngeal carcinoma	5 (1.6)	4 (2.6)	0 (0.0)	1 (1.1)
Rhabdoid tumor	5 (1.6)	2 (1.3)	0 (0.0)	3 (3.2)
Pleuropulmonaal blastoom	1 (0.3)	0 (0.0)	0 (0.0)	1 (1.1)
Other carcinoma	9 (3.0)	4 (2.6)	2 (3.3)	3 (3.2)
Treatment protocol, *n* (%)				
DCOG NBL 2009				
MR	32 (10.5)	11 (7.3)	13 (21.7)	8 (8.5)
HR	85 (27.9)	44 (29.1)	15 (25.0)	26 (27.7)
EURAMOS‐1	42 (13.8)	31 (20.5)	0 (0.0)	11 (11.6)
EWING 2008	4 (1.3)	1 (0.7)	0 (0.0)	3 (3.2)
MAKEI 05	45 (14.8)	23 (15.2)	12 (20.0)	10 (10.6)
PHITT	13 (4.3)	5 (3.3)	5 (8.3)	3 (3.2)
SIOPEL 3	7 (2.3)	1 (0.7)	5 (8.3)	1 (1.1)
SIOPEL 4	8 (2.6)	3 (2.0)	4 (6.7)	1 (1.1)
Wilms SIOP2001	24 (7.9)	10 (6.6)	2 (3.3)	12 (12.8)
UMBRELLA 2016	17 (5.6)	8 (5.3)	2 (3.3)	7 (7.4)
EpSSG‐RMS 2005	5 (1.6)	4 (2.6)	0 (0.0)	1 (1.1)
EpSSg‐NRSTS 2005	5 (1.6)	2 (1.3)	0 (0.0)	3 (3.2)
NPC‐2003 GPOH	5 (1.6)	4 (2.6)	0 (0.0)	1 (1.1)
Other	13 (4.3)	4 (2.6)	2 (3.3)	7 (7.4)
Treatment, *n* (%)				
Cisplatin	180 (59.0)	93 (61.6)	33 (55.0)	54 (57.4)
Carboplatin	53 (17.4)	21 (13.9)	4 (6.7)	28 (29.8)
Cisplatin and carboplatin	52 17.0)	24 (15.9)	21 (35.0)	7 (7.4)
Oxaliplatin	1 (0.3)	0 (0.0)	0 (0.0)	1 (1.1)
Cisplatin oxaliplatin	2 (0.7)	0 (0.0)	0 (0.0)	2 (2.1)
Cisplatin carboplatin and oxaliplatin	2 (0.7)	1 (0.7)	0 (0.0)	1 (1.1)
H&N‐RT	5 (1.6)	5 (3.3)	0 (0.0)	0 (0.0)
H&N‐RT + cisplatin	5 (1.6)	4 (2.6)	0 (0.0)	1 (1.1)
H&N‐RT + ENT surgery	1 (0.3)	1 (0.7)	0 (0.0)	0 (0.0)
ENT surgery + cisplatin	4 (1.3)	2 (1.3)	2 (3.3)	1 (1.1)
Deceased within 1 year after last chemotherapy, *n* (%)	70 (23.0)	17 (11.3)	5 (8.3)	48 (51.1)

Abbreviations: BERA, brainstem‐evoked response audiometry; CPA, conditioned play audiometry; ENT, ear, nose, and throat; H&N‐RT, head and neck radiotherapy; *N* , number; OAE, oto‐acoustic emissions; VRA, visual reinforcement audiometry.

**TABLE 3:** Audiological evaluation summary table.

		Audiological evaluation after end of ototoxic treatment		
		Yes (*N* = 211)		No (*N* = 94)
		CPA/PTA subcohort	Other audiological test (OAE, BERA, VRA) subcohort	
		*N* = 151	*N* = 60	*N* = 94
Audiological evaluation				
Baseline audiogram, *n* (%)	191	96 (63.6)	42 (70.0)	53 (56.4)
Audiogram during treatment, *n* (%)	230	122 (80.8)	51 (85.0)	57 (60.6)
Median time between end of treatment and audiogram in months (range)		2.0 (−2.9–78.8)	1.6 (−11.6–17.2)	−2.6 (−35.2–−0.3)

Abbreviations: BERA, brainstem‐evoked response audiometry; CPA, conditioned play audiometry; *N* , number; OAE, oto‐acoustic emissions; VRA , visual reinforcement audiometry.

### 3.3 Audiological Monitoring

Baseline audiological monitoring was performed in 191 patients (191/305, 62.6%) with 230 patients (230/291, 79.0%) receiving audiological monitoring during treatment and 211 patients (211/257, 82.1%) completed end of treatment assessment. Of these 211 evaluations, 151 included CPA/PTA. The remaining 60 patients were evaluated using VRA, BERA, DPOAE, and/or TEOAE (Figure 1). Audiological monitoring included different tests at baseline and end of treatment according to the patient's age at diagnosis and physical state (Figure S1). In 12.1% of the children (*n* = 37), audiological monitoring was neither performed during nor after treatment, 18.7% (*n* = 57) had audiological monitoring during treatment but not at the end. Reasons for no audiological evaluation at the end of treatment are summarized in Table S3.

### 3.4 Prevalence of HL

As seen in Table 4, of the 211 patients with an audiological examination available after ototoxic treatment, 108 children (51.2%) suffered from HL according to the Muenster criteria (Muenster ≥ 2b audiogram or derivative BERA, VRA/OAE result), 83 patients (39.3%) did not have HL, and 20 children (9.5%) had inconclusive audiological test results (Figure 1). According to the SIOP classification, HL (SIOP ≥ 2 audiogram or derivative BERA, VRA/OAE result) occurred in 77 children (36.5%), 122 children (57.8%) did not suffer from HL, and inconclusive test results were obtained from 12 children (5.7%). 11 patients (7.3%) tested by ear‐specific CPA/PTA had asymmetric HL. HL prevalence in the CPA/PTA and BERA, VRA, OAE subpopulation can be found in Table 4.

**TABLE 4:** Audiological results according to Muenster and SIOP grading at the end of treatment.

Total *n* = 211	Muenster < 2b	Muenster ≥ 2b	Inconclusive	SIOP < 2	SIOP ≥ 2	Inconclusive
	(*n* = 83)	(*n* = 108)	(*n* = 20)	(*n* = 122)	(*n* = 77)	(*n* = 12)
CPA/PTA (*n* = 151)	69 (83.1%)	78 (72.2%)*	4 (20.0%)	98 (80.3%)	49 (63.6%)	4 (33.3%)
OAE, BERA, VRA (*n* = 60)	14 (16.9%)	30 (27.8%)	16 (80.0%)	24 (19.7%)	28 (36.4%)	8 (66.7%)

Abbreviations: BERA, brainstem‐evoked response audiometry; CPA, conditioned play audiometry; *N*, number; OAE, oto‐acoustic emissions; VRA, visual reinforcement audiometry.

* Asymmetry was observed in 11 (11/151, 7.3%) CPA/PTA‐tested patients.

### 3.5 Determinants of HL

Univariate analyses of the total cohort with or without HL at the end of treatment, classified according to Muenster ototoxicity criteria (*n* = 191), showed that age at diagnosis < 5 years, age at diagnosis (continue), total cumulative dose (TCD) furosemide (mg/kg), TCD cisplatin per 100 mg/m^2^, gentamicin, vincristine, vancomycin, and cisplatin treatment were associated with HL (Muenster ≥ 2b or derivative BERA, VRA/OAE test result) (Table 5).

**TABLE 5:** Determinants associated with hearing loss according Muenster at end of treatment: Results of univariable and multivariable logistic regression analyses.

Determinants	No of patients			UVA	MVA*
	Total cohort	Muenster < 2b	Muenster ≥ 2b		
	*N* = 191	*N* = 83	*N* = 108	OR (95% CI)	OR (95%) CI
Sex, *n* (%)					
Male	100 (52.4)	37 (44.6)	63 (58.3)	1.74 (0.98–3.10)	1.93 (0.98–3.79)
Female	91 (47.6)	46 (55.4)	45 (41.7)		
Age at diagnosis					
Median (range)	4.0 (0–18)	7.0 (0–17)	3.5 (0–18)	**0.91 (0.86–0.96), *p* = 0.001**	**0.94 (0.88–1.00), *p* = 0.049**
≤ 5 year	109 (57.1)	37 (44.6)	72 (66.7)		
> 5 year	82 (42.9)	46 (55.4)	36 (33.3)	**0.40 (0.22–0.73), *p* = 0.002**	
Treatment					
Cisplatin					
No	28 (14.7)	22 (26.5)	6 (5.6)		
Yes	163 (85.3)	61 (73.5)	102 (94.4)	**6.13 (2.36–15.96), *p* < 0.001**	
Median TCD mg/m^2^	417.9 (98.7–639.0)	399.3 (98.7–639.0)	467.4 (159.4–635.5)	**1.49 (1.23–1.81) (per 100 mg/m** ^**2**^ **), *p* < 0.001**	**1.64 (1.36–2.00) (per 100 mg/m** ^**2**^ **), *p* < 0.001**
Carboplatin					
No	128 (67.0)	52 (62.7)	76 (70.4)		
Yes	63 (33.0)	31 (37.3)	32 (29.6)	0.71 (0.38–1.30)	
Median TCD mg/m^2^	1513.4 (197.4–5994.6)	2497.4 (197.4–5994.6)	1464.0 (300.0–4907.3)	1.00 (1.00–1.00)	
Oxaliplatin					
No	190 (99.5)	83 (100)	107 (99.1)		
Yes	1 (0.5)	0 (0)	1 (0.9)	NA	
Median TCD mg/m^2^	718.5		718.5		
Vincristine					
No	92 (48.2)	51 (61.4)	41 (38.0)	**2.60 (1.45–4.69), *p* = 0.001**	**3.30 (1.39–7.80), *p* = 0.007**
Yes	99 (51.8)	32 (38.6)	67 (62.0)		
Median TCD mg/m^2^	9.0 (1.3–27.7)	9.1 (1.3–27.7)	8.9 (1.5–17.7)	1.04 (0.99–1.10)	
Gentamicin					
No	149 (76.9)	71 (85.5)	78 (72.2)		
Yes	42 (23.1)	12 (14.5)	30 (27.8)	**2.28 (1.08–4.78), *p* = 0.030**	
Vancomycin					
No	90 (47.1)	52 (62.7)	38 (35.2)		
Yes	101 (52.9)	31 (37.3)	70 (64.8)	**3.09 (1.71–5.60), *p* < 0.001**	1.35 (0.64–2.83)
Teicoplanin					
No	147 (77.0)	68 (81.9)	79 (73.1)		
Yes	44 (23.0)	15 (18.1)	29 (26.9)	1.66 (0.82–3.36)	
Amlodipine					
No	146 (76.4)	64 (77.1)	82 (75.9)		
Yes	45 (23.6)	19 (22.9)	26 (24.1)	1.07 (0.54–2.10)	
Furosemide					
No	120 (62.8)	55 (66.3)	65 (60.2)		
Yes	71 (37.2)	28 (33.7)	43 (39.8)	1.30 (0.72–2.36)	
H&N‐RT					
No	181 (94.8)	79 (95.2)	102 (94.4)		
Yes	10 (5.2)	4 (4.8)	6 (5.6)	NA	
ENT surgery					
No	188 (98.4)	83 (100)	105 (97.2)		
Yes	3 (1.6)	0 (0)	3 (2.8)	NA	

*Note:* Bold indicates statistically significant results (*p* < 0.05).

Abbreviations: CI, confidence interval; ENT, ear‐nose‐throat; H&N‐RT, head and neck radiotherapy; MVA, multivariate analysis; NA, not applicable; OR, odds ratio; PTA, pure‐tone audiometry; TCD, total cumulative dose; UVA, univariate analysis.

* MVA includes sex, age at diagnosis (continue), TCD cisplatin, vincristine, and vancomycin treatment.

Multivariable analyses, including sex, age at diagnosis, TCD cisplatin, vincristine, and vancomycin treatment, revealed that age at diagnosis (OR 0.9, 95% CI 0.9–1.0), the TCD cisplatin per 100 mg/m^2^ (OR 1.6, 95% CI 1.4–2.0) and vincristine (OR 3.3, 95% CI 1.4–7.8) remained significantly associated with HL (Table 5).

We split the Muenster‐based dataset (*n* = 191 observations) into a training set (75%) and a test set (25%). The 10‐fold validation revealed an average accuracy of 0.66 (± 0.14) and an AUC‐ROC of 0.74 (± 0.12). Applying the model to the test data showed an accuracy of 0.68 (95%CI 0.52–0.81). Sensitivity and specificity were respectively 0.82 and 0.56. An additional subanalysis of the CPA/PTA subcohort was performed, and comparable results were obtained (Table S4). The overall accuracy was 0.77 (± 0.06) and AUC‐ROC 0.82 (± 0.12).

An additional subanalysis of the CPA/PTA and cisplatin‐treated subcohort was performed, comparable results were obtained (Tables S5 and S6, Figure S2). Vincristine remained associated with cisplatin‐induced HL (CIHL) (Muenster ≥ 2b) (OR 4.7 95% CI 2.1–10.6) (Table S5).

Univariate analyses of the total cohort, classified according to SIOP ototoxicity criteria (*n* = 199), showed that age ≤ 5 years, age at diagnosis, TCD cisplatin per 100 mg/m^2^, vincristine, male sex, gentamicin, vancomycin, and cisplatin treatment were associated with HL (SIOP ≥ 2 or derivative BERA, VRA/OAE test result) (Table 6).

**TABLE 6:** Determinants associated with hearing loss according SIOP grading at end of treatment: Results of univariable and multivariable logistic regression analyses.

Determinants	No of patients		
	UVA	MVA*
			Total cohort end of treatment monitoring	SIOP < 2	SIOP ≥ 2
*N* = 199	*N* = 122	*N* = 77	OR (95% CI)	OR (95%) CI
Sex, *n* (%)					
Male	104 (52.3)	55 (45.1)	49 (63.6)	**2.13 (1.19–3.83), *p* = 0.011**	**2.69 (1.38–5.27), *p* = 0.004**
Female	95 (47.7)	67 (54.9)	28 (36.4)		
Age at diagnosis					
Median (range)	4.0 (0–18)	6.0 (0–18)	3.0 (0–16)	**0.89 (0.84–0.94), *p* < 0.001**	**0.88 (0.83–0.95), *p* < 0.001**
≤ 5 year	116 (58.3)	57 (46.7)	59 (76.6)		
> 5 year	83 (41.7)	65 (53.3)	18 (23.4)	**0.27 (0.14–0.51), *p* < 0.001**	
Treatment					
Cisplatin					
No	29 (14.6)	27 (22.1)	2 (2.6)		
Yes	170 (85.4)	95 (77.9)	75 (97.4)	**10.66 (2.46–46.26), *p* = 0.002**	
Median TCD mg/m^2^	405.3 (98.7–639.0)	400.0 (98.7–639.0)	456.8 (157.8–635.5)	**1.49 (1.23–1.81)** **(per 100 mg/m** ^**2**^ **), *p* < 0.001**	**1.49 (1.23–1.81)** **(per 100 mg/m** ^**2**^ **), *p* < 0.001**
Carboplatin					
No	133 (66.8)	82 (67.2)	51 (66.2)		
Yes	66 (33.2)	40 (32.8)	26 (33.8)	1.05 (0.57–191)	
Median TCD mg/m^2^	1515.3 (197.4–5994.6)	2197.7 (197.4–5994.6)	1152.8 (300.0–4907.3)	1.00 (1.00–1.00)	
Oxaliplatin					
No	198 (99.5)	122 (100)	76 (98.7)		
Yes	1 (0.5)	0 (0)	1 (1.3)	NA	
Median TCD mg/m^2^	718.5		718.5		
Vincristine					
No	96 (48.2)	66 (54.1)	30 (39.0)		
Yes	103 (51.8)	56 (45.9)	47 (61.0)	**1.85 (1.03–3.30), *p* = 0.038**	0.82 (0.37–1.84)
Median TCD mg/m^2^	8.9 (1.3–27.7)	9.0 (1.3–27.7)	8.9 (3.0–12.9)	1.02 (0.97–1.08)	
Gentamicin					
No	156 (78.4)	102 (83.6)	54 (70.1)		
Yes	43 (21.6)	20 (16.4)	23 (29.9)	**2.17 (1.10–4.31), *p* = 0.026**	
Vancomycin					
No	94 (47.2)	69 (56.6)	25 (32.5)		
Yes	105 (52.8)	53 (43.4)	52 (67.5)	**2.71 (1.49–4.92), *p* = 0.001**	0.62 (0.29–1.32)
Teicoplanin					
No	152 (76.4)	97 (79.5)	55 (71.4)		
Yes	47 (23.6)	25 (20.5)	22 (28.6)	1.55 (0.80–3.01)	
Amlodipine					
No	151 (75.9)	94 (77.0)	57 (74.0)		
Yes	48 (24.1)	28 (23.0)	20 (26.0)	1.18 (0.61–2.28)	
Furosemide					
No	126 (63.3)	82 (67.2)	44 (57.1)		
Yes	73 (36.7)	40 (32.8)	33 (42.9)	1.54 (0.85–2.77)	
H&N‐RT					
No	189 (95.0)	114 (93.4)	75 (97.4)		
Yes	10 (5.0)	8 (6.6)	2 (2.6)	NA	
ENT surgery					
No	195 (98.0)	119 (97.5)	76 (98.7)		
Yes	4 (2.0)	3 (2.5)	1 (1.3)	NA	

*Note:* Bold indicates statistically significant results (*p* < 0.05).

Abbreviations: CI, confidence interval; ENT, ear‐nose‐throat; H&N‐RT, head and neck radiotherapy; MVA, multivariate analysis; NA, not applicable; OR, odds ratio; PTA, pure‐tone audiometry; TCD, total cumulative dose; UVA, univariate analysis.

* MVA includes sex, age at diagnosis (continue), TCD cisplatin, vincristine and vancomycin treatment.

Multivariable analyses including sex, age at diagnosis, TCD cisplatin, vincristine and vancomycin revealed that age at diagnosis (OR 0.9, 95% CI 0.8–1.0), TCD cisplatin per 100 mg/m^2^ (OR 1.5, 95% CI 1.2–1.8), and male sex (OR 2.7, 95% CI 1.4–5.3) were significantly associated with HL.

## 4 Discussion

This study evaluated ototoxicity monitoring in a national cohort of 305 patients with solid tumors treated in the Máxima between 2015 and 2020. Our study showed that the majority of children treated with platinum agents, H&N‐RT, or ENT‐surgery had received audiological monitoring at baseline, during treatment, and at the end of therapy (see Figure 1 and Table 3). HL graded according to Muenster and SIOP occurred in 51.2% and 36.5%, respectively, of patients at the end of treatment and was significantly associated with age at diagnosis, male sex, cisplatin, and vincristine treatment.

As compared to our study, similar observations for audiological evaluation have been reported in a previous study by Weiss et al. (2018) [17], who studied HL monitoring among Swiss childhood patients with cancer. The authors concluded that 66% of patients were tested at baseline and 72% after completion of therapy. Both studies observed somewhat fewer audiological assessments at baseline as compared to the end of treatment assessments. An explanation might be the fact that children often need to start cancer treatment as soon as possible, indicating that limited time is available to schedule an audiological evaluation. In addition, critical illness, requiring surgery or intensive care unit admission may prohibit audiological testing at baseline. Nevertheless, if possible, an audiological assessment before start of treatment is important to perform to assess possibility of pre‐existing HL and serves as a reference for comparison of follow‐up test results.

Abnormal hearing after ototoxic cancer therapy was present in about 50% of the children in our national cohort, a percentage that is comparable to previous ototoxicity studies. Moke et al. (2021) [14] revealed that 43.8% of cisplatin‐treated patients had HL (SIOP grade ≥ 2) at latest follow‐up. Perilongo et al. (2009) [22] studied HL in patients with hepatoblastoma and found 32% HL (Brock ≥ 1), Landier et al. (2014) [23] investigated hearing function in patients with high‐risk neuroblastoma and concluded that the prevalence of HL ranged from 8%–71% based on ototoxicity grading scale (CTCAEv3, Brock and Chang) and treatment group. Clemens et al. (2019) [24] compared these ototoxicity grading scales by classifying 3799 audiograms of platinum‐treated patients and found the percentage of HL between 48.2% and 40.5% according to Muenster and SIOP scales, respectively. Overall, the use of different ototoxicity grading systems may lead to different prevalence rates of HL. Nevertheless, it seems that Muenster classification detected HL earliest in time as a Muenster 2b grade exists when thresholds are greater than 40 dB at 8 kHz, whereas an SIOP grade of > 2 HL is achieved when thresholds are greater than 20 dB at 4 kHz [24]. So, an increase of HL for 6 and 8 kHz alone is detected using the Muenster grading and not by the SIOP grading [28] which only detects progression of HL across frequencies. This explains the difference in HL percentages between Muenster and SIOP grading in our cohort. However, it is highly plausible that the prevalences of HL would be closer if the SIOP grading had used a cutoff at grade 1. The slight variation in prevalence of HL between studies could also be a reflection of the large heterogeneity regarding patient characteristics, treatment including different platinum components, dosing schedules, supportive care medication and duration of follow‐up [5]. It is important to note that our study focused on patients with HL > grade 2, as ototoxicity < grade 2 could not be assessed due to the lack of high‐frequency data for all subjects suggesting that additional cases of mild HL may not have been identified. The high percentages of HL found in both the current study and previous studies highlight the importance of performing audiological evaluations within 3 months after end of treatment in all patients at risk for ototoxicity.

In our study, DPOAEs and TEOAEs were mainly performed in children ≤ 5 years. The use of OAEs is recommended in young children to test cochlear function, as they do not require a conscious response to a sound stimulus. Especially the use of DPOAE is important to screen the higher frequencies (up to 10 kHz) for signs of early impairment in cochlear status. The use of these tests, however, remains limited to screening purposes only, as in contrast to PTA their results cannot be graded [18]. Currently, no clear cutoff point is available to determine normal or abnormal hearing function according to DPOAEs, and it is well known that the test conditions, including calibration of the equipment [25], background noise, and clinical conditions that affect middle ear status such as otitis media with effusion and cerumen impaction [26, 27], can influence the test. Additional behavioral audiological testing is important to confirm abnormal OAE results. There is an unmet need for international consensus on interpreting OAE results in young children treated with ototoxic medication.

In children older than 5 years of age, information on hearing status is mostly obtained through PTA and is preferably accompanied by EHF‐PTA, as recommended by experts [18]. In the current study, however, additional EHF‐PTA was not always performed. EHF‐PTA is especially important for early detection of ototoxicity because it is well established that cochlear damage due to platinum containing chemotherapy starts in the high frequencies [28]. If possible, EHF‐PTA is therefore recommended to be performed at baseline and throughout follow‐up for early detection of HL.

In terms of middle ear assessment, we found that tympanometry was not always performed as part of audiological monitoring. Furthermore, otoscopy was never performed as a standard test. Otoscopy and tympanometry are important to determine the status of the ear canal and middle ear, especially to rule out middle ear pathology. Given our inability to consistently assess the impact of middle ear pathology/conductive HL in this cohort, we acknowledge that conductive HL may have influenced the results of this study. Experts recommend the use of tympanometry and otoscopy to accompany every audiological evaluation, especially in the case of an abnormal result [18].

Our study found that cisplatin TCD was associated with HL occurrence. These findings are in accordance with previous literature [10, 15, 29, 30, 31]. For example, Wei et al. (2019) [29] studied TCD cisplatin in patients with solid tumor and showed that for each unit growth of accumulated cisplatin dose the probability to develop HL increased by 1.004 (95% CI, 1.001–1.006). Another study found that an increase of 100 mg/m^2^ results in a hazard ratio of 1.2 (95% CI: 1.2–1.5) for HL in cisplatin‐treated childhood cancer survivors [15]. Therefore, it is highly recommended to screen children, who receive treatment with high doses of cisplatin, frequently for HL. Furthermore, three recently published retrospective studies concluded that vincristine exposure was significantly associated with cisplatin‐induced HL [14, 15, 31]. However, Riga et al. 2005 [32], who prospectively studied the effect of vincristine on OAEs in a cohort of leukemia patients, found no significant alterations in OAEs. In addition, Lugassy et al. (1996) [33] concluded that there was no evidence for the ototoxic effect of vincristine in a prospective cohort of patients with lymphoproliferative malignancies, evaluated for HL by PTA. To date, the pathophysiological mechanism of vincristine‐induced HL remains unclear. Therefore, more prospective research is needed on the contribution of vincristine on HL development, especially in cases of multimodal cancer treatment.

Young age at diagnosis was associated with HL development, in line with previous studies that reported this observation [30, 34, 35, 36]. A possible explanation might be the influence of cancer treatment on the still developing peripheral and central auditory pathways, especially in infants and younger children [37, 38]. As these children are in their early speech and language development, timely detection of HL is important to start hearing rehabilitation early if needed [7].

We also found that male sex was associated with HL. This observation was also made by Yancey et al. (2012) [30] in a study of 102 pediatric patients who were treated with cisplatin for osteosarcoma, neuroblastoma, hepatoblastoma, or germ cell tumor (mean age at diagnosis was 8.7 years). A possible reason for this finding is that there were more male patients among diagnoses with high rates of ototoxicity such as liver tumors and osteosarcoma, while the group of germ cell tumors (in which less ototoxicity was observed) was predominantly female. Other studies could not find a significant association between sex and HL development [15, 39, 40]. More research in larger patient cohorts with the same disease and treatment is necessary to investigate this association further.

This study has multiple strengths. First, it is a cohort study including all patients with solid tumors treated between 2015 and 2020 in the Netherlands. The Máxima and Wilhelmina Children's Hospital have the facilities that allow for comprehensive audiological testing of children of all ages. Second, the audiologists who assessed the hearing tests have considerable experience with ototoxicity monitoring and can therefore make adequate estimations of the child's hearing status. Our study also has some limitations. In retrospect, hearing tests were not always repeated when a test result was inconclusive. We were therefore not able to interpret the hearing status of all children at the end of treatment. Furthermore, abnormal tympanograms were taken into account if available; however, tympanometry was not always performed which may have affected the outcome of our study, as negative middle ear pressure can result in transient conductive HL [41]. Due to the low number of participants treated with H&N‐RT and ENT surgery, it was not possible to perform an in depth analysis on the association with HL development. Finally, information on ototoxic treatment was retrospectively retrieved from patient files. Sufficient data were obtainable on the TCD of cisplatin, carboplatin, and vincristine, but not for aminoglycosides, glycopeptides, and loop diuretics, as supportive care medication can also be prescribed by general practitioners or shared care medical centers. The association between comedication and HL occurrence should therefore be interpreted with caution and further investigated.

## 5 Conclusion

In conclusion, most children underwent audiological monitoring during and at the end of ototoxic treatment. HL occurred in up to 50% of the children after completion of ototoxic treatment. The findings of this study confirm once again the importance of audiological monitoring in childhood cancer patients at risk for ototoxicity. The use of age‐appropriate test batteries is highly recommended at standardized time points, including measurements at baseline, during and after end of cancer treatment [42]. Uniform grading is highly necessary to compare interpatient and intrapatient test results. This will lead to timely recognition of HL and provides the opportunity to offer counseling and to apply (audiological) interventions if necessary. Improved insight into clinical determinants allows for identification of children at high risk for ototoxicity, who may benefit from prevention strategies that are currently being developed and implemented.

## Author Contributions

**Franciscus A. Diepstraten:** conceptualization (equal), data curation (equal), formal analysis (equal), methodology (equal), project administration (equal), visualization (equal), writing – original draft (equal). **Odette M. M. Bertram:** project administration (equal), resources (equal), writing – review and editing (equal). **Hiske W. Helleman:** investigation (equal), supervision (equal), validation (equal), writing – review and editing (equal). **Ralf A. Boerboom:** investigation (equal), supervision (equal), validation (equal), writing – review and editing (equal). **Martine van Grotel:** investigation (equal), supervision (equal), validation (equal), writing – review and editing (equal). **József Zsíros:** investigation (equal), supervision (equal), validation (equal), writing – review and editing (equal). **Godelieve A. M. Tytgat:** investigation (equal), supervision (equal), validation (equal), writing – review and editing (equal). **Harm van Tinteren:** formal analysis (equal), methodology (equal), supervision (equal), validation (equal), visualization (equal), writing – review and editing (equal). **Robert J. Stokroos:** conceptualization (equal), investigation (equal), methodology (equal), supervision (equal), writing – review and editing (equal). **Geert O. Janssens:** investigation (equal), supervision (equal), validation (equal), writing – review and editing (equal). **Alex E. Hoetink:** conceptualization (equal), investigation (equal), methodology (equal), resources (equal), supervision (equal), validation (equal), writing – review and editing (equal). **Marry M. van den Heuvel‐Eibrink:** conceptualization (equal), investigation (equal), methodology (equal), resources (equal), supervision (equal), validation (equal), writing – review and editing (equal). **Annelot J. M. Meijer:** conceptualization (equal), data curation (equal), formal analysis (equal), investigation (equal), methodology (equal), project administration (equal), resources (equal), supervision (equal), validation (equal), writing – review and editing (equal).

## Ethics Statement

The study was conducted according to the guidelines of the Declaration of Helsinki, and approved by the Institutional Review Board of the Princess Máxima Center for Pediatric Oncology (PMCLAB2021.275).

## Consent

The Biobank and Data Access Committee of the Princess Máxima Center for Pediatric Oncology decided that treatment‐related data and audiological data were obtained during regular treatment and follow‐up visits and could be collected via the Biobank informed consent form.

## Conflicts of Interest

The authors declare no conflicts of interest.

## Supporting information


**Table S1.** Current childhood cancer protocols in which ototoxic treatments and medication are frequently administered.
**Table S2.** Ototoxicity classification systems.
**Table S3.** Reasons for no audiological test at the end of treatment.
**Table S4.** Determinants associated with hearing loss according Muenster at end of treatment: results of univariable and multivariable logistic regression analyses CPA/PTA subcohort.
**Table S5.** Determinants associated with cisplatin‐induced hearing loss according to Muenster grading at end of treatment: results of univariable and multivariable logistic regression analyses.
**Table S6.** Determinants associated with hearing loss according SIOP at end of treatment: results of univariable and multivariable logistic regression analyses CPA/PTA subcohort.
**Figure S1.** Audiological tests at baseline and end of ototoxic treatment.
**Figure S2.** CIHL cohort (*N* = 163) probability of hearing loss by continuous variables.

## Data Availability

The data that support the findings of this study are available on request from the corresponding author. The data are not publicly available due to privacy or ethical restrictions.
