# Supplementary material for: A Retrospective Evaluation of Ototoxicity Monitoring in a Cohort of Pediatric Patients With Solid Tumors, Treated in the Dutch National Cancer Center
Source: Cancer Rep (Hoboken). 2024 Nov 26;7(11):e70046. doi: 10.1002/cnr2.70046 (PMC11590333; doi:10.1002/cnr2.70046)
Supplement: Supplementary file 1 — Table S1. Current childhood cancer protocols in which ototoxic treatments and medication are frequently administered. Table S2. Ototoxicity classification systems. Table S3. Reasons for no audiological test at the end of treatment. Table S4. Determinants associated with hearing loss according Muenster at end of treatment: results of univariable and multivariable logistic regression analyses CPA/PTA subcohort. Table S5. Determinants associated with cisplatin‐induced hearing loss according to Muenster grading at end of treatment: results of univariable and multivariable logistic regression analyses. Table S6. Determinants associated with hearing loss according SIOP at end of treatment: results of univariable and multivariable logistic regression analyses CPA/PTA subcohort. Figure S1. Audiological tests at baseline and end of ototoxic treatment. Figure S2. CIHL cohort (N = 163) probability of hearing loss by continuous variables. [file CNR2-7-e70046-s001.docx]

**Supplement content**

Supplementary Table 1. Current childhood cancer protocols in which ototoxic treatments and medication are frequently administered (page 2).

Supplementary Table 2. Ototoxicity classification systems (page 3).

Supplementary Table 3. Reasons for no audiological test at the end of treatment (page 4).

Supplementary Table 4. Determinants associated with hearing loss according Muenster at end of treatment: results of univariable and multivariable logistic regression analyses CPA/PTA subcohort (page 5-6).

Supplementary Table 5. Determinants associated with cisplatin-induced hearing loss according to Muenster grading at end of treatment: results of univariable and multivariable logistic regression analyses (page 7-8).

Supplementary Table 6. Determinants associated with hearing loss according SIOP at end of treatment: results of univariable and multivariable logistic regression analyses CPA/PTA subcohort (page 9-10).

Supplementary Figure 1. Audiological tests at baseline and end of ototoxic treatment (page 11).

Supplementary Figure 2 . CIHL cohort (N=163) Probability of hearing loss by continuous variables (page 12).

References (page 13).

| **SUPPLEMENTARY TABLE 1.** Current childhood cancer protocols in which ototoxic treatments and medication are frequently administered. | | | | | | |
| --- | --- | --- | --- | --- | --- | --- |
| **Protocol** | **Dx** | **Cisplatin** | **Carboplatin** | **H&N-RT** | **Medication*** | **Recommendations for hearing loss surveillance** |
| DCOG NBL 2009 (1) | NBL | X | X |  | X | MRG: audiogram before 1st, 2nd and 3rd N5  HRG: audiometry before 1st MIBG, before 1st and 2nd N5 and before start of ASCT.  Additionally, an audiogram at the end of therapy. |
| EURAMOS-1(2) | OS | X |  |  | X | Pre-treatment audiogram, before the 3rd and 4th cycle, and after the last cycle. |
| MAKEI 05 (3) | GCT | X |  |  | X | One pre-treatment audiogram and one audiogram before each cycle |
| PHITT (4) | HB, HCC | X | X |  | X | Pre-treatment audiogram before 1st chemotherapy cycle, at the end of therapy, and annual assessments thereafter. |
| SIOPEL 4 (5) | HB | X | X |  | X | Audiogram at diagnosis, before cycle A2 and cycle A3, in week 3 of block B, pre-operative, at the end of therapy and yearly until reliable result obtained with pure tone audiometry age 3 years+ |
| SIOPEL 3 (6) | HB, HCC | X | X |  | X | Audiogram at diagnosis, prior to surgery, at the end of therapy and yearly until reliable result obtained with pure tone audiometry age 3 years+ |
| NPC-2003 GPOH (7) | NPC | X |  | X | X | An audiogram should be performed at diagnosis. |
| UMBRELLA 2016 (8) | REN |  | X |  | X | Post-operative audiogram in week 1 and 10, and at end of therapy. |
| SIOP2001 (9) | WT |  | X |  | X | This protocol contains no recommendations for hearing loss surveillance. |
| EpSSG-RMS 2005 (10) | RMS |  | X | X | X | Audiological assessment once a year in case of cochlear irradiation. |
| EpSSG-NRSTS 2005 (11) | NRSTS |  | X | X | X | Audiological assessment once a year in case of cochlear irradiation. |
| EWING 2008 (12) | ES |  |  | X | X | This protocol contains no recommendations for hearing loss surveillance. |
| Adapted from Meijer et al., JAMA Oncol. 2021.(Meijer et al., 2021)  Abbreviations: ASCT=autologous stem cell transplantation; Dx=diagnosis; ES=Ewing sarcoma; GCT=germ cell tumor; HB=hepatoblastoma; HCC=hepatocellular carcinoma; H&N-RT= head and neck radiotherapy; NBL=neuroblastoma; NPC=nasopharyngeal carcinoma; NRSTS=non-rhabdomyosarcoma soft tissue sarcoma; OS=osteosarcoma; REN=renal tumor; RMS=rhabdomyosarcoma; WT=Wilmstumor.  *Includes glycopeptides, aminoglycosides, and/or loop diuretics’ | | | | | | |

| **SUPPLEMENTARY TABLE 2.** Ototoxicity classification systems | | | | |
| --- | --- | --- | --- | --- |
| **SIOP Grade** | **SIOP** | **Muenster grade** | **Muenster** | **Deleterious hearing loss** |
| 0 | ≤ 20 dB HL at all frequencies | 0 | ≤10 dB HL at all frequencies | No |
| 1 | > 20 dB HL SNHL* at > 4 kHz | 1 | >10 dB HL ≤20 dB HL at any frequency |  |
|  |  | 2a | >20 dB HL ≤ 40 dB HL at ≥4 kHz |  |
| 2 | > 20 dB HL SNHL at 4 kHz and above |  |  | Yes |
|  |  | 2b | >40 dB HL ≤ 60 dB HL at ≥4 kHz |  |
|  |  | 2c | >60 dB HL at ≥4 kHz |  |
| 3 | > 20 dB HL SNHL at 2 or 3 kHz and above | 3a | >20 dB HL ≤ 40 dB HL at <4 kHz |  |
|  |  | 3b | >40 dB HL ≤ 60 dB HL at <4 kHz |  |
|  |  | 3c | >60 dB HL< 80 dB HL at <4 kHz |  |
| 4 | > 40 dB HL SNHL at 2 kHz and above | 4 | ≥80 dB at <4 kHz |  |
| * Hearing loss due to a pathology of the cochlea, auditory nerve, or central nervous system.  Abbreviations: dB=Decibel, HL= hearing loss, kHz=kilohertz | | | | |

| **SUPPLEMENTARY TABLE 3.** Reasons for no audiological test at the end of treatment | |
| --- | --- |
|  | **N=94** |
| **No audio during and at the end of treatment** | **N=37** |
| **Reasons**  Deceased shortly after start treatment  No recommendation to audiology in the treatment protocol  No clear reason documented | N=14  N=10  N=13 |
| **Audio during treatment but not at the end of treatment** | **N=57** |
| **Reasons**  Deceased during or shortly after treatment  No recommendation to audiology in the treatment protocol  Logistic reasons  External audiological follow-up  Multiple relapses  No clear reason | N=34  N=5  N=1  N=1  N=1  N=15 |

| **SUPPLEMENTARY TABLE 4.** Determinants associated with hearing loss according to Muenster grading at end of treatment: results of univariable and multivariable logistic regression analyses CPA/PTA subcohort. | | | | | |
| --- | --- | --- | --- | --- | --- |
|  | No of patients | | | UVA  OR (95% CI) | MVA*  OR (95% CI) |
| **Determinants** | Total CPA/ PTA sub-cohort  N=147 | **Muenster <2b**  **N=69** | **Muenster ≥2b**  **N=78** |  |  |
| Sex, n (%)  Male  Female | 78 (53.1)  69 (46.9) | 30 (43.5)  39 (56.5) | 48 (61.5)  30 (38.5) | **2.08 (1.08-4.02)** | 1.95 (0.93-4.08) |
| Age at diagnosis  Median (range)  ≤5yr  >5yr | 7.0 (0-18)  65 (44.2)  82 (55.8) | 10.0 (0-17)  23 (33.3)  46 (66.7) | 5.0 (0-18)  42 (53.8)  36 (46.2) | **0.91 (0.85-0.97)**  **0.43 (0.22-0.84)** | 0.70 (0.28-1.75) |
| Treatment |  |  |  |  |  |
| - Cisplatin   No  Yes  Median TCD mg/m^2^ | 25 (17.0)  122 (83.0)  470.1 (98.7-639.0) | 20 (29.0)  49 (71.0)  404.0(98.7-639.0) | 5 (6.4)  73 (93.6)  475.5 (159.4-600.0) | **5.96 (2.10-16.94)**  **1.49 (1.23-1.81)**  **(per 100 mg/m^2^)** | **1.49 (1.23-1.81)**  **(per 100 mg/m^2^)** |
| - Carboplatin   No  Yes  Median TCD mg/m^2^ | 103 (70.1)  44 (29.9)  1698.9 (197.4-5089.3) | 44 (63.7)  25 (36.2)  2539.4 (197.4-5089.3) | 59 (75.6)  19 (24.4)  1465.9 (300.0-3982.7) | 0.57 (0.28-1.16)  1.0 (0.999-1.00) |  |
| - Oxaliplatin   No  Yes  Median TCD mg/m^2^ | 146 (99.3)  1 (0.7)  718.5 | 69 (100)  0 (0) | 77 (98.7)  1 (1.3)  718.5 | NA |  |
| - Vincristine   No  Yes  Median TCD mg/m^2^ | 69 (46.9)  78 (53.1)  9.0 (1.3-27.7) | 40 (58.0)  29 (42.0)  9.2 (1.3-27.7) | 29 (37.2)  49 (62.8)  8.9 (1.5-17.7) | **2.33 (1.20-4.52)**  1.02 (0.97-1.09) | **2.96 (1.09-8.05)** |
| - Gentamicin   No  Yes | 113 (76.9)  34 (23.1) | 57 (82.6)  12 (17.4) | 56 (71.8)  22 (28.2) | 1.87 (0.84-4.13) |  |
| - Vancomycin   No  Yes | 72 (49.0)  75 (51.0) | 43 (62.3)  26 (37.6) | 29 (37.2)  49 (62.8) | **2.79 (1.43-5.46** | 1.28 (0.56-2.92) |
| - Teicoplanin   No  Yes | 112 (76.2)  35 (23.8) | 57 (82.6)  12 (17.4) | 55 (70.5)  23 (29.5) | 1.99 (0.90-4.38) |  |
| - Amlodipine   No  Yes | 113 (76.9)  34 (23.1) | 56 (81.2)  13 (18.8) | 57 (73.1)  21 (26.9) | 1.59 (0.73-3.48) |  |
| - Furosemide   No  Yes | 87 (59.2)  60 (40.8) | 44 (63.8)  25 (36.2) | 43 (55.1)  35 (44.9) | 1.43 (0.74-2.78) |  |
| - H&N-RT   No  Yes | 137 (93.2)  10 (6.8) | 65 (94.2)  4 (5.8) | 72 (92.3)  6 (7.7) | NA |  |
| - ENT-surgery   No  Yes | 144 (98.0)  3 (2.0) | 69 (100)  0 (0) | 75 (96.2)  3 (3.8) | NA |  |
| Abbreviations: CI, confidence interval; CPA, conditioned play audiometry; ENT, ear-nose-throat; H&N-RT; head and neck radiotherapy, MVA, multivariate analysis; NA, not applicable; OR, odds ratio; PTA, pure tone audiometry; TCD, total cumulative dose; UVA, univariate analysis.  * MVA includes: sex, age≤ 5 yrs/>5 yrs, TCD cisplatin, vincristine and vancomycin treatment. | | | | | |

| **SUPPLEMENTARY TABLE 5.** Determinants associated with cisplatin-induced hearing loss according to Muenster grading at end of treatment: results of univariable and multivariable logistic regression analyses | | | | | |
| --- | --- | --- | --- | --- | --- |
|  | No of patients | | | UVA  OR (95% CI) | MVA*  OR (95% CI) |
| **Determinants** | Total cisplatin treated cohort  N=163 | **Muenster <2b**  **N=61** | **Muenster ≥2b**  **N=102** |  |  |
| Sex, n (%)  Male  Female | 85 (52.1)  78 (47.9) | 25 (29.4)  36 (46.2 | 60 (70.6)  42 (53.8) | **2.06 (1.08-3.92)** |  |
| Age at diagnosis  Median (range)  ≤5yr  >5yr | 4.0 (0.0-18.0)  90  73 | 12.0 (0.0-17.0)  22 (24.4)  39 (53.4) | 3.5 (0.0-18.0)  68 (75.6)  34 (46.6) | **0.90 (0.85-0.95)**  **3.55 (1.82-6.90)** | 0.95 (0.89-1.01) |
| Treatment |  |  |  |  |  |
| - Cisplatin   Median TCD mg/m^2^ | 417.9 (98.7-639.0) | 399.3 (98.7-639.0 | 467.4 (159.4-635.5) | **1.35 (1.11-1.64)**  **per 100 mg/m^2^** | **1.34 (0.96-1.74)**  **per 100 mg/m^2^** |
| - Carboplatin   No  Yes  Median TCD mg/m^2^ | 122 (74.8)  41 (25.2)  1354.7 (300.0-3920.1) | 49 (40.2)  12 (29.3)  1205.1 (400.0-3009.9) | 73 (59.8)  29 (70.7)  1409.6 (300.0-3920.1) | 1.62 (0.76-3.48)  1.00 (1.00-1.00) |  |
| - Oxaliplatin   No  Yes  Median TCD mg/m^2^ | 162 (99.4)  1 (0.6)  718.5 | 61 (37.7)  0 (0.0) | 101 (62.4)  1 (100.0)  718.5 | NA  NA |  |
| - Vincristine   No  Yes  Median TCD mg/m^2^ | 90 (55.2)  73 (44.8)  8.9 (1.3-16.6) | 49 (54.4)  12 (16.4)  9.0 (1.3-9.2) | 41 (45.6)  61 (83.6)  8.9 (1.5-16.6) | **6.08 (2.88-12.80)**  **1.25 (1.13-1.37)** | **4.66 (2.05-10.59)** |
| - Gentamicin   No  Yes | 122 (74.8)  41 (25.2) | 50 (41.0)  11 (26.8) | 72 (59.0)  30 (73.2) | 0.53 (0.24-1.51) |  |
| - Vancomycin   No  Yes | 72 (44.2)  91 (55.8) | 39 (54.2)  22 (24.2) | 33 (45.8)  69 (75.8) | **3.71 (1.90-7.22)** |  |
| - Teicoplanin   No  Yes | 121 (74.2)  42 (25.8) | 48 (39.7)  13 (31.0) | 73 (60.3)  29 (69.0) | 1.47 (0.69-3.10) |  |
| - Amlodipine   No  Yes | 126 (77.3)  37 (22.7) | 49 (38.9)  12 (32.4) | 77 (61.1)  25 (67.6) | 1.33 (0.61-2.88) |  |
| - Furosemide   No  Yes | 98 (60.1)  65 (39.9) | 39 (39.8)  22 (33.8) | 59 (60.2)  43 (66.2) | 1.29 (0.67-2.48) |  |
| - H&N-RT   No  Yes | 159 (97.5)  4 (2.5) | 60 (37.7)  1 (25.0) | 99 (62.3)  3 975.0) | NA |  |
| - ENT-surgery   No  Yes | 161 (98.8)  2 (1.2) | 61 (37.9)  0 | 100 (62.1)  2 (100.0) | NA |  |
| Abbreviations: ENT, ear-nose-throat; H&N-RT, head and neck radiotherapy; MVA, Multivariate analysis; NA, not applicable; OR, odds ratio; TCD, total cumulative dose; UVA, univariate analysis.  * MVA includes: age at diagnosis (continue), TCD cisplatin and vincristine | | | | | |

| **SUPPLEMENTARY TABLE 6.** Determinants associated with hearing loss according to SIOP grading at end of treatment: results of univariable and multivariable logistic regression analyses CPA/PTA subcohort. | | | | | |
| --- | --- | --- | --- | --- | --- |
|  | No of patients | | | UVA  OR (95% CI) | MVA*  OR (95% CI) |
| **Determinants** | Total CPA/ PTA sub-cohort  N=147 | **SIOP<2**  **N=98** | **SIOP ≥2**  **N=49** |  |  |
| Sex, n (%)  Male  Female | 78 (53.1)  69 (46.9) | 44 (44.9)  54 (55.1) | 34 (69.4)  15 (30.6) | **2.78 (1.35-5.75)** | **3.25 (1.40-7.54)** |
| Age at diagnosis  Median (range)  ≤5yr  >5yr | 7.0 (0-18)  65 (44.2)  82 (55.8) | 10.0 (0-17)  33 (33.7)  65 (66.3) | 5.0 (0-18)  32 (65.3)  17 (34.7) | **0.88 (0.82-0.94)**  **0.27 (0.13-0.56)** | **0.23 (0.081-0.65)** |
| Treatment |  |  |  |  |  |
| - Cisplatin   No  Yes  Median TCD mg/m^2^ | 25 (17.0)  122 (83.0)  470.1 (98.7-639.0) | 24 (24.5)  74 (75.5)  457.0 (98.7-639.0) | 1 (2.0)  48 (98.0)  473.8 (157.8-600.0) | **15.57 (2.04-118.90)**  **1.35 (1.11-1.64)**  **(per 100 mg/m^2^)** | **27.26 (3.17-234.44)** |
| - Carboplatin   No  Yes  Median TCD mg/m^2^ | 103 (70.1)  44 (29.9)  1698.9 (197.4-5089.3) | 68 (69.4)  30 (30.6)  2518.4 (197.4-5089.3) | 35 (71.4)  14 (28.6)  808.5 (300-2525.2) | 0.91 (0.43-1.93)  1.00 (1.00-1.00) |  |
| - Oxaliplatin   No  Yes  Median TCD mg/m^2^ | 146 (99.3)  1 (0.7)  718.5 | 98 (100)  0 (0) | 48 (98.0)  1 (2.0)  718.5 | NA |  |
| - Vincristine   No  Yes  Median TCD mg/m^2^ | 69 (46.9)  78 (53.1)  9.0 (1.3-27.7) | 52 (53.1)  46 (46.9)  9.1 (1.3-27.7) | 17 (34.7)  32 (65.3)  9.0 (3.0-12.9) | **2.13 (1.05-4.33)**  1.02 (0.96-1.09) | 1.43 (0.49-4.18) |
| - Gentamicin   No  Yes | 113 (76.9)  34 (23.1) | 81 (82.7)  17 (17.3) | 32 (65.3))  17 (34.7) | **2.53 (1.15-5.56)** |  |
| - Vancomycin   No  Yes | 72 (49.0)  75 (51.0) | 57 (58.2)  41 (41.8) | 15 (30.6)  34 (69.4) | **3.15 (1.52-6.53)** | 1.40 (0.55-3.56) |
| - Teicoplanin   No  Yes | 112 (76.2)  35 (23.8) | 79 (80.6)  19 (19.4) | 33 (67.3)  16 (32.7) | 2.02 (0.93-4.40) |  |
| - Amlodipine   No  Yes | 113 (76.9)  34 (23.1) | 81 (82.7)  17 (17.3 | 32 (65.3)  17 (34.7) | **2.5 (1.15-5.56)** |  |
| - Furosemide   No  Yes | 87 (59.2)  60 (40.8) | 63 (64.3)  35 (35.7) | 24 (49.0)  25 (51.0) | 1.88 (0.94-3.76) |  |
| - H&N-RT   No  Yes | 137 (93.2)  10 (6.8) | 90 (91.8)  8 (8.2) | 47 (95.9)  2 (4.1) | NA |  |
| - ENT-surgery   No  Yes | 144 (98.0)  3 (2.0) | 95 (96.9)  3 (3.1) | 49 (100)  0 (0) | NA |  |
| Abbreviations: CI, confidence interval; CPA, conditioned play audiometry; ENT, ear-nose-throat; H&N-RT, head and neck radiotherapy; MVA, Multivariate analysis; NA, not applicable; OR, odds ratio; PTA, pure tone audiometry; UVA, univariate analysis.  * MVA model includes: sex, age≤ 5 yrs/>5 yrs,, cisplatin, vincristine and vancomycin treatment. | | | | | |

**SUPPLEMENTARY FIGURE 1.** Audiological tests at baseline and end of ototoxic treatment. Abbreviations: DPOAE=distortion product otoacoustic emissions; TEOAE= transiently evoked otoacoustic emissions; BERA=Brainstem Evoked Response Audiometry; VRA=Visual Reinforcement Audiometry; CPA=Conditioned Play Audiometry; PTA=Pure Tone Audiometry; EHF=Extended-High Frequency; SA=Speech Audiometry. A combination of multiple audiological tests may have been performed per patient. Age at start treatment.

**SUPPLEMENTARY FIGURE
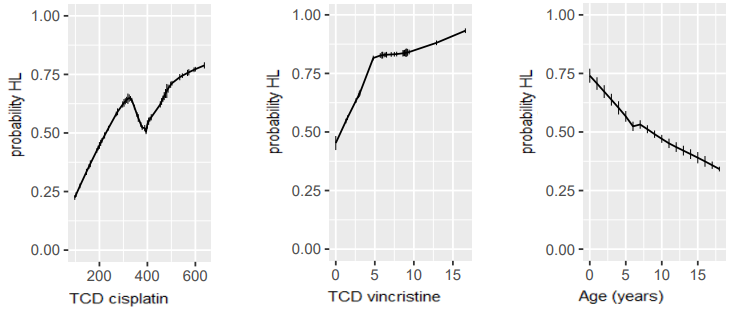
2**. CIHL cohort (N=163) Probability of hearing loss by continuous

variables. Abbreviations: HL, Hearing Loss; TCD, Total Cumulative Dose in mg/ m^2^.

**References**

1. Dutch Childhood Oncology Group D. DCOG NBL 2009 TREATMENT PROTOCOL for Risk Adapted Treatment of Children with Neuroblastoma. . 2012.

2. Whelan JS, Bielack SS, Marina N, Smeland S, Jovic G, Hook JM, et al. EURAMOS-1, an international randomised study for osteosarcoma: results from pre-randomisation treatment. Ann Oncol. 2015;26(2):407-14.

3. German Society of Pediatric Oncology and Hematology G. MAKEI 05: Prospective Trial for the diagnosis and treatment of children, adolescents and young adults with Extracranial Germ Cell Tumors including Sex Cord Stromal Tumors and small cell ovarian carcinoma, hypercalcemic type. 2005.

4. PHITT. PHITT. Paediatric Hepatic International Tumour Trial: PHITT. University of Birmingham 2018.

5. Zsiros J, Brugieres L, Brock P, Roebuck D, Maibach R, Zimmermann A, et al. Dose-dense cisplatin-based chemotherapy and surgery for children with high-risk hepatoblastoma (SIOPEL-4): a prospective, single-arm, feasibility study. Lancet Oncol. 2013;14(9):834-42.

6. Oncology. ISOP. SIOPEL - 3 Liver Tumour Studies: Hepatoblastoma And Hepatocellular Carcinoma. International Society Of Paediatric Oncology.; 1998.

7. Buehrlen M, Zwaan CM, Granzen B, Lassay L, Deutz P, Vorwerk P, et al. Multimodal treatment, including interferon beta, of nasopharyngeal carcinoma in children and young adults: preliminary results from the prospective, multicenter study NPC-2003-GPOH/DCOG. Cancer. 2012;118(19):4892-900.

8. Vujanić GM, Gessler M, Ooms A, Collini P, Coulomb-l'Hermine A, D'Hooghe E, et al. The UMBRELLA SIOP-RTSG 2016 Wilms tumour pathology and molecular biology protocol. Nat Rev Urol. 2018;15(11):693-701.

9. Committee. SNTaS. SIOP WILMS 2001 study protocol. 2001.

10. Bisogno G, Jenney M, Bergeron C, Gallego Melcón S, Ferrari A, Oberlin O, et al. Addition of dose-intensified doxorubicin to standard chemotherapy for rhabdomyosarcoma (EpSSG RMS 2005): a multicentre, open-label, randomised controlled, phase 3 trial. Lancet Oncol. 2018;19(8):1061-71.

11. Ferrari A, van Noesel MM, Brennan B, Zanetti I, Corradini N, Casanova M, et al. Paediatric non-rhabdomyosarcoma soft tissue sarcomas: the prospective NRSTS 2005 study by the European Pediatric Soft Tissue Sarcoma Study Group (EpSSG). Lancet Child Adolesc Health. 2021;5(8):546-58.

12. groups EaNAEss. EWING 2008. protocol version 2.0, December 2015.
